# Effect of *Rhizoglomus fasciculatum* and *Paecilomyces lilacinus* in the biocontrol of root-knot nematode, *Meloidogyne incognita* in *Capsicum annuum* L

**DOI:** 10.1080/19420889.2021.2025195

**Published:** 2022-03-07

**Authors:** Bhoopander Giri, Renuka Rawat, Geeta Saxena, Preet Manchanda, Qiang-Sheng Wu, Anuradha Sharma

**Affiliations:** a Department of Botany, Swami Shraddhanand College, University of Delhi, Delhi, India; b Department of Botany, University of Delhi, Delhi, India; c College of Horticulture and Gardening, Yangtze University, Jingzhou, China; d Department of Botany, Hindu College, University of Delhi, Delhi, India

**Keywords:** Arbuscular mycorrhiza, nematodes, egg parasite, biocontrol, soil amendment, nematophagous fungi

## Abstract

Root-knot nematodes possess a major threat to agricultural production of various crops worldwide. The intensive use of chemical nematicides to control plant parasitic nematodes has adverse effects on our environment and human health. Owing to the importance of developing new strategies, an experiment was conducted to reveal the influence of arbuscular mycorrhizal fungus, *Rhizoglomus fasciculatum* and nematophagous fungus, *Paecilomyces lilacinus* alone or in combination with various organic amendments such as superphosphate, green and organic manure to control the infection of root-knot, nematode *Meloidogyne incognita* in a vegetable crop *Capsicum annuum*. These two fungi along with soil amendments significantly improved plant growth and fruit yield and effectively controlled infection of *M. incognita*. The dual inoculation of *P. lilacinus* and *R. fasciculatum* reduced the number of galls and egg masses, thereby revealing the controlled proliferation of *M. incognita* infection in *C. annuum* roots. The beneficial effect of these fungi further increased on supplementation of soil with organic or green manures. Inoculation of *C. annuum* with these two fungi showed a significant increase in egg parasitization; however, maximum effect was detected on dual inoculation. Amongst the soil amendments, the best response was obtained in case of green manure along with mycorrhizal fungus and *P. lilacinus*. Present study revealed that nematophagous and AM fungi, in combination with green manure were effective in controlling *M. incognita*, thus suggesting the use of such agents for biocontrol of plant parasitic nematodes in agriculture.

## Introduction

Plant parasitic nematodes, the unseen enemies, are a diverse group of obligate pathogens obtaining nutrition from the cytoplasm of living cells of the host. All major field crops, most vegetables, certain cash crops, ornamental plants and many weeds or grasses are susceptible to one or more species of root-knot nematodes [1]. An estimate of $157 billion has been attributed to the damages occurred by plant parasitic nematodes [2], which is more significant than the damage caused by invasive insects, which is approximately $70 billion [3]. The full extent of worldwide nematode damage is likely to be miscalculated, since growers are often unaware of their presence because the symptoms caused in the plant are often nonspecific [4,5].

Nematodes are usually small soil-borne pathogens feeding on the different parts of a plant via roots, stems, leaves, flowers and seeds, although most species feed on roots [6]. They feed through a protrusible stylet that penetrates the plant cells [4,7]. Based on their feeding habitats, plant-parasitic nematodes are categorized as either ectoparasitic or endoparasitic. Sedentary endoparasitic nematodes consist of the root-knot (*Meloidogyne* spp.) and cyst nematodes (*Heterodera* and *Globodera* spp.), which are the most common nematodes in terms of crop losses [4].

Root-knot nematodes disrupt the normal processes of plant root growth and its soil exploration capacity for both water and nutrients; consequently, the infected plant shows above ground symptoms, such as stunting, yellowing, excessive wilting and reduced yield [8].

So far, several strategies have been developed for the control of nematode in agriculture [9]; however, strategies associated with biocontrol are proposed as a much safer alternative and highly practicable for plant-parasitic nematodes management [10]. *Paecilomyces lilacinus* has been proven as an effective potential biocontrol agent for root-knot and cyst nematodes under both greenhouse and field conditions [11]. *P. lilacinus* displays nematophagous capacity through different mechanisms of action, traps several fungi and phytopathogenic bacteria, as well as acts as bio-stimulants [11–13]. *P. lilacinus* treated plants exhibit reduced root galling with increased plant health and crop yield in the soils infested by root–knot nematodes [14]. Inoculation of plants with *P. lilacinus* results in the reduction in the hatching of egg masses as well as the final juvenile population of nematodes in soil [15].

Interaction between *M. incognita* and arbuscular mycorrhizal fungi showed promising results against nematode pathogens, but application of arbuscular mycorrhizal fungi for biocontrol of plant pathogens remains insignificant. Recent literature suggests that the contribution of mycorrhizal fungi through mycorrhiza-mediated enhancement of plant nutrition, competition with the pathogen for resources and space, plant morphological changes and barrier formation, changes in biochemical compounds are related with plant resistance response, alleviation of physical stresses and changes in antagonist and deleterious microbes’ population in the mycorrhizosphere features biological control of root borne plant pathogens [16].

Soil amendments mainly manures, crop residues, composts, oil cakes, ash, animal and human wastes are by products and wastes from agriculture or other activities. The nematicidal effects of some of these soil amendments have been recognized for some time and reviewed extensively [17–18]. Addition of organic amendments to the soil stimulates microbial activity as evidenced by increased population of algae, nematode-trapping fungi, bacteria, microbivores nematodes, and other soil antagonists that destroy plant parasitic nematodes [20]. Proliferation of microorganisms results in increased enzymatic activities of the amended soil. They promote decomposition of residues resulting in the soils of specific compounds that could be nematicidal [8,19].

Several members of Solanaceae including, *Capsicum annuum* are affected by root-knot nematode attack and severely reducing their yields worldwide. The chemical control of root-knot nematodes has not been effective; therefore, an alternative strategy to overcome the problem of nematodes management could be use of biocontrol agents. Hence, the present study aimed to understand the effect of nematophagous fungus *Paecilomyces lilacinus* and mycorrhizal fungus, *Rhizoglomus fasciculatum* alone or in combination along with different soil amendments in the biocontrol of root-knot nematode *M. incognita.*

## Materials and methods

### Plants and soil materials

The vegetable crop plant *Capsicum annuum* var. California Wonder belonging to family Solanaceae was selected as experimental plant and their seeds were procured from NBPGR, Indian Agricultural Research Institute, New Delhi. The garden soil and sand (2:1) was mixed properly, hereafter referred as soil. The soil was air-dried, passed through a 2-mm sieve and analyzed by preparing an extract as suggested by 21. The soil had the following properties: loam texture; pH (H_2_O) 7.5, EC 1.0 (dS/m), Organic C (%) 1.15, Total N (%) 0.49, Available P and K 11.1 and 55 mg kg^−1^, respectively. Soil was sterilized thoroughly using 0.1% formaldehyde (Excelar, Qualigen) and covered with black polythene for a week and eventually exposed for aeration. The process was repeated twice. Earthenware pots of size 28 cm × 25 cm (4.5 kg capacity) were filled with this sand-soil potting mixture. Seedlings were raised in the same soil. After 4 weeks, five seedlings were transplanted in each pot.

### Inoculum production

**Figure 1.**
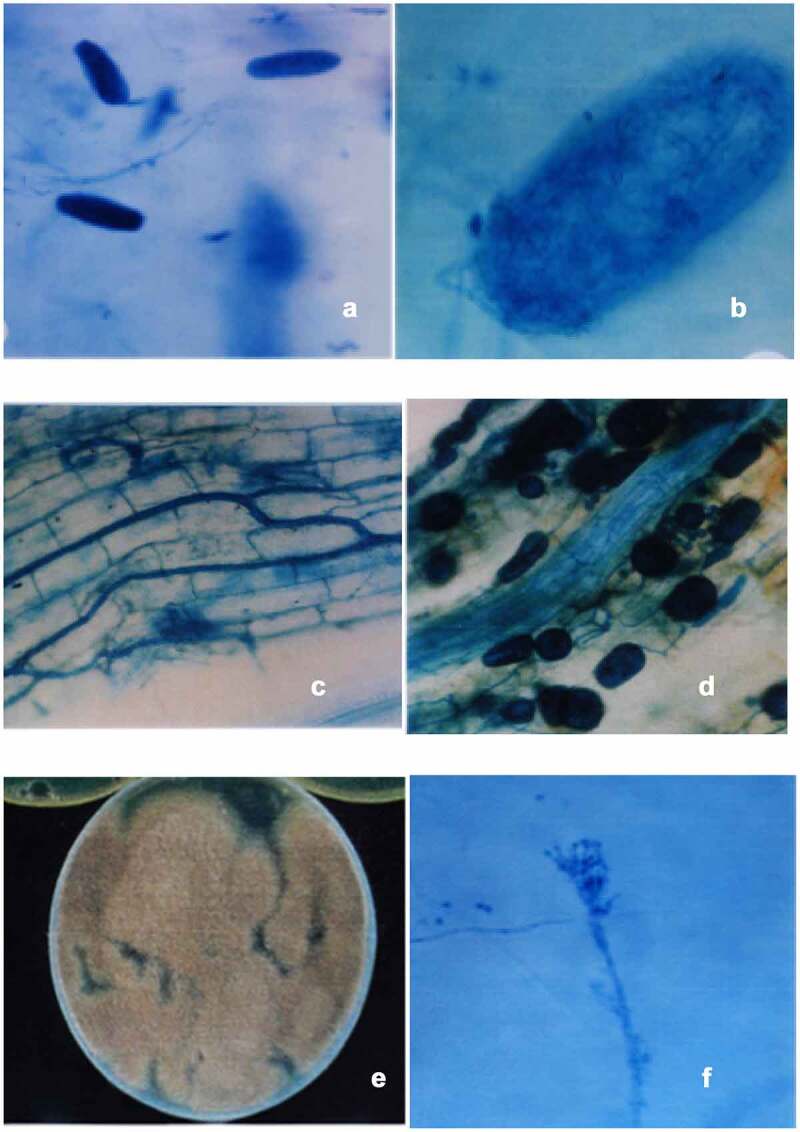
Eggs of *M. incognita* with fungal mycelium (a &b); Colonization of *R. fasciculatum* in the root cortical cells of *C. annuum* (c) and showing variously shaped intracellular vesicles of *R. fasciculatum* (d); Isolated egg parasite from rhizosphere of *Capsicum annuum* (e); Conidiophore of *Paecilomyces lilacinus* showing conidia in chain(f).

The pure culture of pathogen *M. incognita* Kofoid and White was obtained from the Department of Nematology, Indian Agriculture Research Institute, Delhi, maintained and multiplied on the roots of Brinjal cv Pusa purple long. Inoculum was prepared according to the method of Hussey and Barker’s [22] and the eggs of nematodes were isolated from infected roots and made to hatch for producing inoculum [Figure 1a].

Arbuscular Mycorrhizal fungus, *Rhizoglomus fasciculatum* (Thaxt.) Sieverd., G.A. Silva & Oehl comb. nov. (Syn. *Glomus fasciculatum* (Thaxt.) Gerd. and Trappe, *Rhizophagus fasciculatus* (Thaxt.) C. Walker and A. Schüßler) was obtained from our laboratory culture collection, which was isolated from the soil of botanical garden of University of Delhi, India. The soil-based culture of *R. fasciculatum* was maintained and multiplied on *Sorghum bicolor* and *Trigonella foenum-graecum* in garden soil, alternatively. The inoculum consisted of thoroughly mixed rhizosphere soil samples containing spores, hyphae and mycorrhizal root fragments [Figure 1 b, c & d]. To standardize the number of spores per gram soil, spores were isolated with a wet sieving and decanting method [23] and counted. A 100 g of soil containing 140 AM fungal spores was added to each pot. Non-Mycorrhizal plants received the same quantity of non-inoculated soil to ensure the same microflora.

*Paecilomyces lilacinus* (Thom.) Samson was maintained and multiplied on Potato Dextrose Agar (PDA) at 26 ± 2°C. For bulk inoculum, nine different agro-based carrier substrates were tried, out of which Chicken feed + Sand (3:1) and Rice bran + rice (2:1) were used in the present investigation. The 100 g of Chicken feed + Sand was placed in each of twenty-five 250 ml Erlenmeyer flasks and autoclaved for 15 min at 121°C, 15 psi. Spore suspension of fungus was prepared by adding sterile distilled water to the 7-days-old culture, and agar surface was gently rubbed to release the fungal spores [Figure 1 e & f]. The 5 ml of spore suspension was used to inoculate cooled, autoclaved carrier substrate present in each flask. The infested carrier substrate was incubated at 26 ± 2°C for 21 days resulting in a fungal inoculum level of about 4.7 × 10^8^/g of substrate. Chicken feed medium was used as carrier substrate. Similar procedure was employed to inoculate carrier substrate Rice bran + rice and fungal inoculum level of 4.5 × 10^8^/g of substrate was raised. A 20 g of inoculated carrier substrate was added to each pot. Non-fungus treatments received equal amounts of non-inoculated carrier substrate to ensure similar soil conditions.

### Soil amendments

To evolve an efficient and inexpensive cultural practice (soil amendment) due to its nematicidal properties and not just to reduce nematode menace but also to enhance effectivity of biocontrol agent and to improve the soil health, three different soil amendments were tested in the present study. Their type, application rate and time of application are presented in Table 1.

**Table 1.** Fungal treatments supplemented with different soil amendments

S N.	Soil amendments	Application rate	Time of application
1	Superphosphate	28 g/kg	applied (3 week) before transplanting at the depth of 10–12 cm
2	Organic Manure (Khadi commission)	44.4 g/kg	applied (3 week) before transplanting and thoroughly mixed, soil was watered
3	Green manure (residue of *Medicago sativa* and *Sesbania aegyptiaca*)	88.8 g/kg	applied (3 week) before transplanting

### Experimental setup

Experiment with five treatments lasting 60 days (after nematode inoculation) was carried out. Following treatments were employed: (1) Control (C) (2) N (nematode *Meloidogyne incognita*) (3) N + F (NF, *M. incognita* with *P. lilacinus*) (4) N + M(NM, *M. incognita* with *R. fasciculatum*) (5) N + M + F (*M. incognita* (N) + *R. fasciculatum* (M) + *P. lilacinus* (F) (NMF), with the following soil amendments (a) Control (Cs, without amendment), (b) Superphosphate (Sp), (c) Organic manure (OM) (commercially prepared containing 0.7% N, 0.15% P and 0.4% K), (d) Green manure (GM) (leaves, and tender shoots were collected and finely chopped, weighed and then added to each pot. NPK composition in % dry weight C 38–40, N 4–9, P 0.69 and K 1.22).

Each treatment was repeated 5 times in a factorial design. Pots were planted with 4-week-old *Capsicum annuum* seedlings which were allowed to establish for 1 week before nematode juveniles were pipetted with water into holes made around the roots. The soil amendments were incorporated earlier (3 weeks) to transplanting. Two fungi, *Paecilomyces lilacinus* and *Rhizoglomus fasciculatum* were added simultaneously during transplantation alone or in combination with nematodes.

### Harvest and analysis

Plants were harvested at the end of 60 days (5 replicates per treatment), which potentially allowed for the development of at least two life cycles of nematodes. Plants were dug out carefully, washed to get free of soil without damaging the roots and screened. Root and shoot were measured, and dry weights of shoot and root were taken separately after oven drying when the stable weight was achieved. Mature fruits were harvested and weighed in flushes. Total fruit yield per treatment was calculated.

To assess root knot infection, plant roots were carefully washed under tap water and were stained using sodium hypochlorite acid fuchsin method [24]. Numbers of galls/root system and egg masses/root system were determined by visual observation. Ten egg masses were randomly collected from infected roots and each egg mass was squashed in a drop of 0.01% sodium hypochlorite solution and observed under 45x and quashed 100x. The presence of fungal mycelium in the egg masses was taken as an indicator of confirmed infection. Infected egg masses were examined to estimate the number of eggs destroyed. Presence of fungal hyphae and spores inside eggs and vacuolation of embryos were taken as a measure of egg destruction. Apparently, healthy eggs were also counted. Empty eggs were presumed to be hatched, escaping infection, therefore counted as healthy eggs for the purpose of computing percent eggs parasitized. The percent parasitization was determined according to the formula given by 25. Three such readings were taken and average percent parasitization was calculated.

On termination of the experiment, *P. lilacinus* was isolated from the soil. Soil samples were taken from each treatment with fungus amendment and stored in plastic vials in a refrigerator for a week and air dried at room temperature for 24 h. Stock was prepared by adding 10 g soil to 100 ml sterile distilled water which was further diluted to 10^3^. The 1 ml of aliquot of the dilution was transferred to each of four petri plates followed by 15 ml of cooled *P. lilacinus* semi-selective medium in each of the petri plate. The petri plates were gently swirled to distribute the sample in the medium. Petri plates were incubated at 25 ± 2°C for 10 days. At harvest, after shoots were cut at the crown, roots were washed carefully under running tap water. Roots were cleared with 10% KOH and stained with 0.1% trypan blue (w/v) in lactophenol as described by [26]. The roots were cut into small segments of 1 cm length. About 100 segments were mounted and observed under stereomicroscope (MAGNUS) to examine mycorrhizal colonization. A root segment was considered to be mycorrhizal, showing presence of mycorrhizal fungal hyphae. Percent mycorrhizal colonization in roots was calculated following Nicolson’s formula, where percent root colonization is equal to the number of segment with mycorrhizal/total number of segments) × 100.

### Statistical analysis

Treatment effects were determined by one-way analysis of variance (ANOVA) using a factorial design. Differences between the treatments were confirmed by Duncan’s Multiple range test (DMRT). A significant level of 0.05 was applied.

## Results

### Fungal colonization

**Figure 2.**
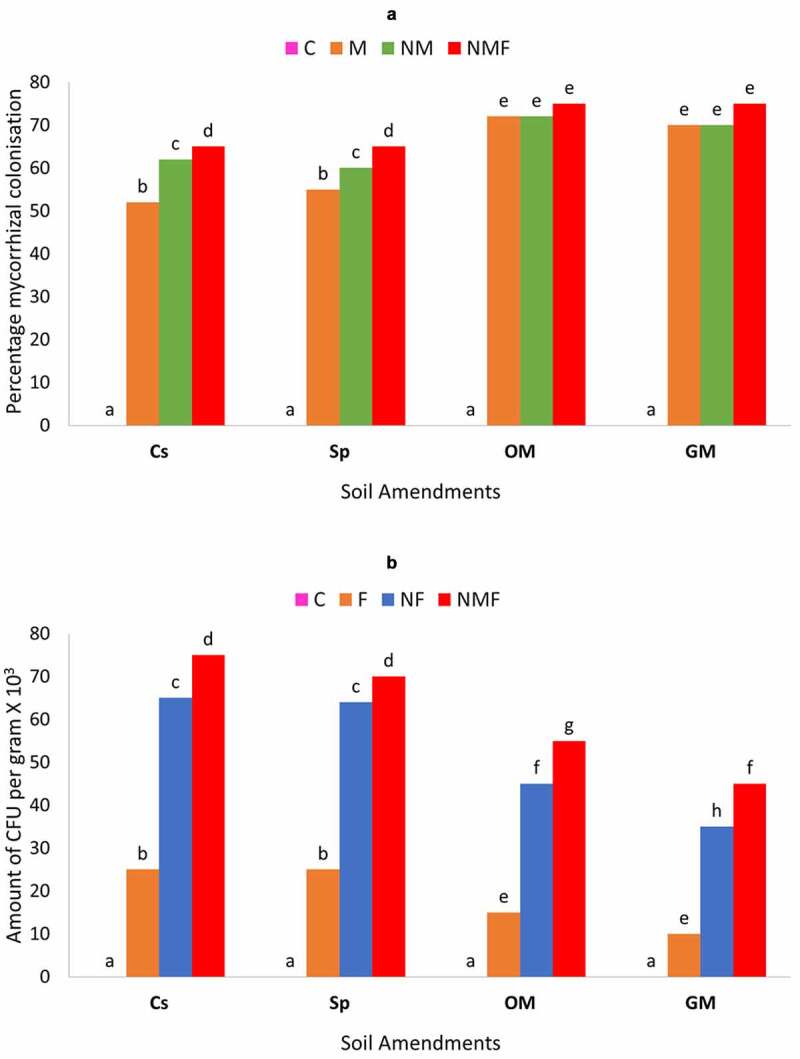
Effect of different soil amendments and treatments alone or in combinations on the percentage of mycorrhizal colonization (a), amount of CFU per gram X 10^3^ (b) of *C. annuum* plants infected with *M. incognita*. Within columns values of each parameter with the same superscript are not significantly different at (*P* = 0.05) Duncan’s multiple range test (DMRT). Soil amendments, Cs – Control Set, Sp – Superphosphate, GM – Green Manure, OM – Organic Manure, and Soil treatments, C – Control, M – *R. fasciculatum*, NM – *M. incognita* with *R. fasciculatum*, NMF – *M. incognita* with *R. fasciculatum* and *P. lilacinus.*

Plant growth parameters were observed at the end of 60 days to examine the effect of different treatments alone or in combination in respect to different soil amendments. Furthermore, the effect of plant parasitic nematode, *M. incognita* and different soil amendments was studied on the percent colonization of arbuscular mycorrhizal fungus, *Rhizoglomus fasciculatum* and amount of CFU/g by *P. lilacinus* [Figure 2 a & b] in the rhizosphere of *C. annuum* plants. The highest mycorrhizal colonization was detected in the soil amended with organic and green manure. An opposite trend was observed in the colony formation of *P. lilacinus*. The colonization of *P. lilacinus* was higher in the case of control and superphosphate amendments and lower in GM and OM amendments. The effect of dual inoculation was greater to both the cases, which showed that the presence of *R. fasciculatum* along with *P. lilacinus* and *M. incognita* significantly supported both mycorrhizal or fungal colonization.

### Effect on growth parameters

In the present study, the influence of mycorrhizal fungus, *R. fasciculatum* and *P. lilacinus* was rectilinear on the growth of *C. annuum* grown in *M. incognita* infected soil, amended with green manure, organic matter and superphosphate. The inoculation of *C. annuum* plants with mycorrhizal fungus and *P. lilacinus* either alone or in combination showed a significant increase in root and shoot length under all types of soil amendments as compared to the uninoculated control plants infected by *M. incognita* [Figure 3a & b]. The *M. incognita* parasitization indeed had a negative impact on plant shoot and root lengths; however, the inoculation with mycorrhizal fungus *R. fasciculatum* and *P. lilacinus* significantly lessened the damage caused by nematode, and had a positive impact on root and shoot lengths of plant under all soil amendments. Noteworthy was that plants inoculated with the dual inoculum of both fungi (NMF) had highest root and shoot length and showed up to 35.72% increase over nematode (N) infected plants. Among soil amendments, the best growth of root and shoot length was recorded in case of green manure followed by organic manure and superphosphate; however, the latter showed least biocontrol effect [Figure 3a & b].

The effect of nematode infection and fungal colonization was observed on the root and shoot fresh weight of *C. annuum* plants. Both the fungi had a profound effect on the root and shoot fresh weight of *C. annuum* under all types of soil amendments; however, the amendment of soil with green manure and organic manure showed greater increase in the root and shoot fresh weight than that of the soil amended with superphosphate. Compared to the control set, the fresh weight of root and shoot was higher in case of superphosphate but lower than the GM and OM soil amendments. Root and shoot fresh weight significantly increased in plants inoculated with *R. fasciculatum* and *P. lilacinus* alone or in combination. The dual inoculation of both the fungi showed the highest increase in root and shoot fresh weight [Figure 3c & d].

The deteriorating effect of *M. incognita* was also observed on root and shoot dry biomass. Root and shoot dry biomass of *C. annuum* significantly reduced due to *M. incognita* infection (in case of OM (25.34%) and in case of Sp (66.87%) [Figure 3e & f] in comparison to the uninfected healthy plants, but this decrease in plant biomass significantly controlled on inoculation of *C. annuum* plants with *R. fasciculatum* and *P. lilacinus* either alone or in combination. The best increment in the root (up to 22.38%) and shoot dry biomass (up to 57.10% Sp) was recorded in case of NMF treated plants in all soil amendments. In all the cases, infected plants inoculated with *R. fasciculatum* (up to 49.72% for shoot and 13.4% for root) showed better biocontrol effect than *P. lilacinus* (up to 32.60% for shoot and 3.60% for root) treated plants in terms of dry biomass. This observation significantly varies along all soil amendments, in fact, organic and green manure exhibited greater effect on diminishing the impact of *M. incognita* as compared to control set or superphosphate soil amendments.

The effect of *R. fasciculatum* and *P. lilacinus* inoculation alone or in combination was studied on the number of galls per root system [Figure 4a] formed by parasitic nematodes. The number of galls per root system was significantly reduced by the dual inoculation of *C. annuum* with *R. fasciculatum* and *P. lilacinus*. The *R. fasciculatum* colonized plants showed significantly similar biocontrol effect to the plants inoculated with *P. lilacinus* in all soil amendments, except organic manure. Soil with green manure and organic manure had a lower number of galls per root system than the soil with superphosphate, which showed a higher number of galls/root system.

The two fungi exhibited synergistic effect on fruit yield/plant; however, maximum effect was detected on the dual inoculation (NMF) of *C. annuum* plants grown in green and organic manure supplemented as compared to control set and superphosphate supplemented soils [Figure 4d]. The *M. incognita*-infected plants colonized by *R. fasciculatum* showed similar biocontrol effect in terms of fruit yield to infected plants inoculated with *P. lilacinus*.

The *M. incognita* infection showed a sharp increase in the number of egg masses of nematodes per root system irrespective of all soil amendments; however, the highest egg masses were detected in the control (Cs) set [Figure 4b], which gradually decreased in the case of Sp, OM and GM soil amendments. The number of egg masses significantly reduced when *C. annuum* plants were treated with combined inoculum of *R. fasciculatum* and *P. lilacinus* (NMF). In fact, *M. incognita*-infected plants colonized with *R. fasciculatum* alone resulted in a similar number of egg masses, to the infected plants treated with *P. lilacinus*, thereby showing a similar biocontrol effect.

Egg parasitization was maximum in NMF-treated plants [Figure 4c]. The *C. annuum* plants growing in the green manure amended soil showed higher egg parasitization than that of the other soil amendments, although this difference was insignificant when compared to organic manure soil amendment. There was a very marginal difference in egg parasitization between infected plants colonized with *R. fasciculatum* and infected plants treated with *P. lilacinus* alone. However, the NM- and NF-treated plants showed significantly less egg parasitization as compared to the NMF-treated plants.

**Figure 3.**
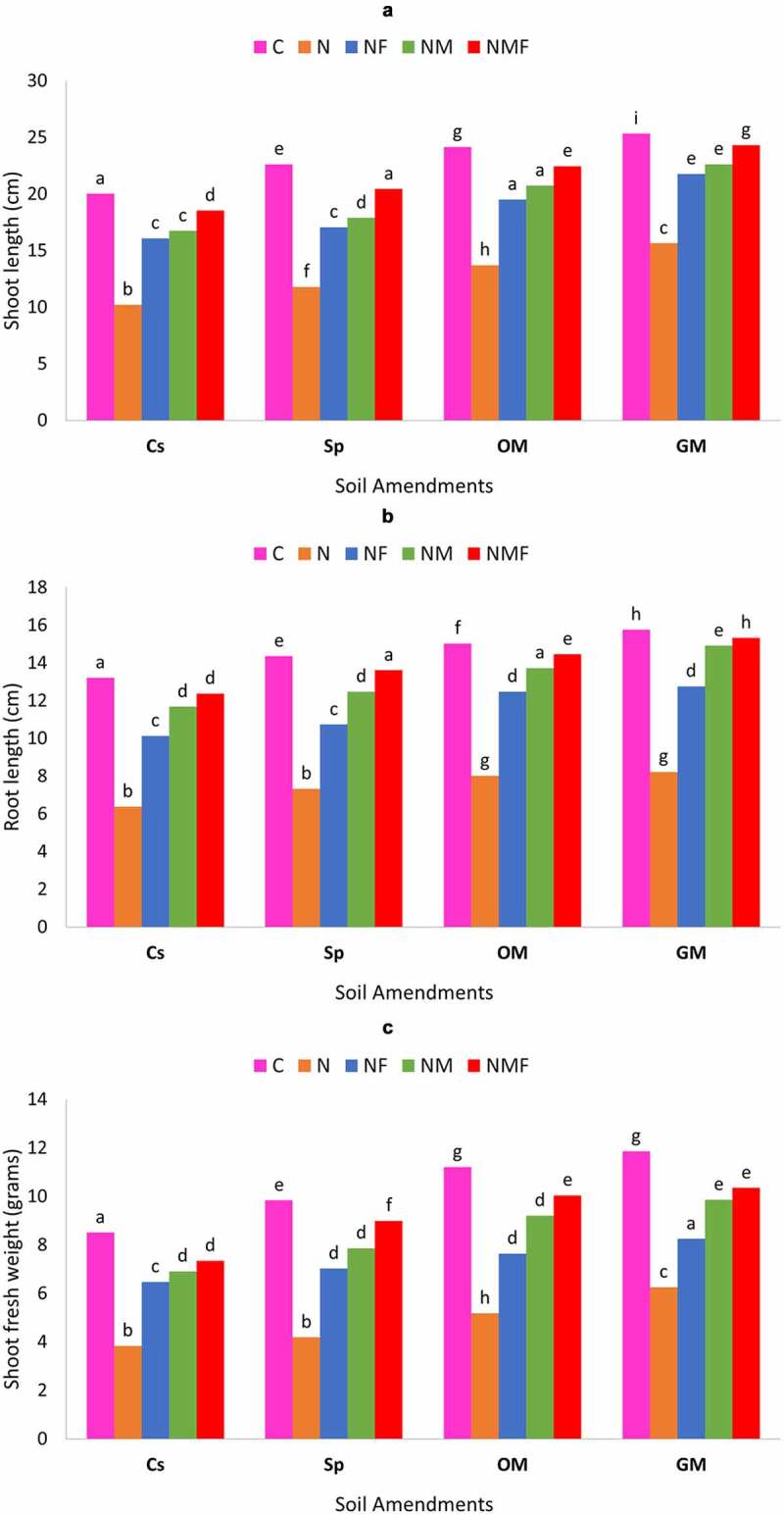
Effect of different treatments and soil amendments alone or in combination on the shoot length (a), root length (b), shoot fresh weight (c), root fresh weight (d), shoot dry weight (e) and root dry weight (f) of *C. annuum* plants infected with *M. incognita*. Within columns values of each parameter with the same superscript are not significantly different at (*P* = 0.05) Duncan’s multiple range test (DMRT). Soil amendments, Cs – Control Set, Sp – Superphosphate, GM – Green Manure, OM – Organic Manure, and Soil treatments, C – Control, N – *M. incognita*, NF – *M. incognita* with *P. lilacinus*, NM – *M. incognita* with *P. lilacinus*, NMF – *M. incognita* with *R. fasciculatum* and *P. lilacinus.*

**Figure 3.**
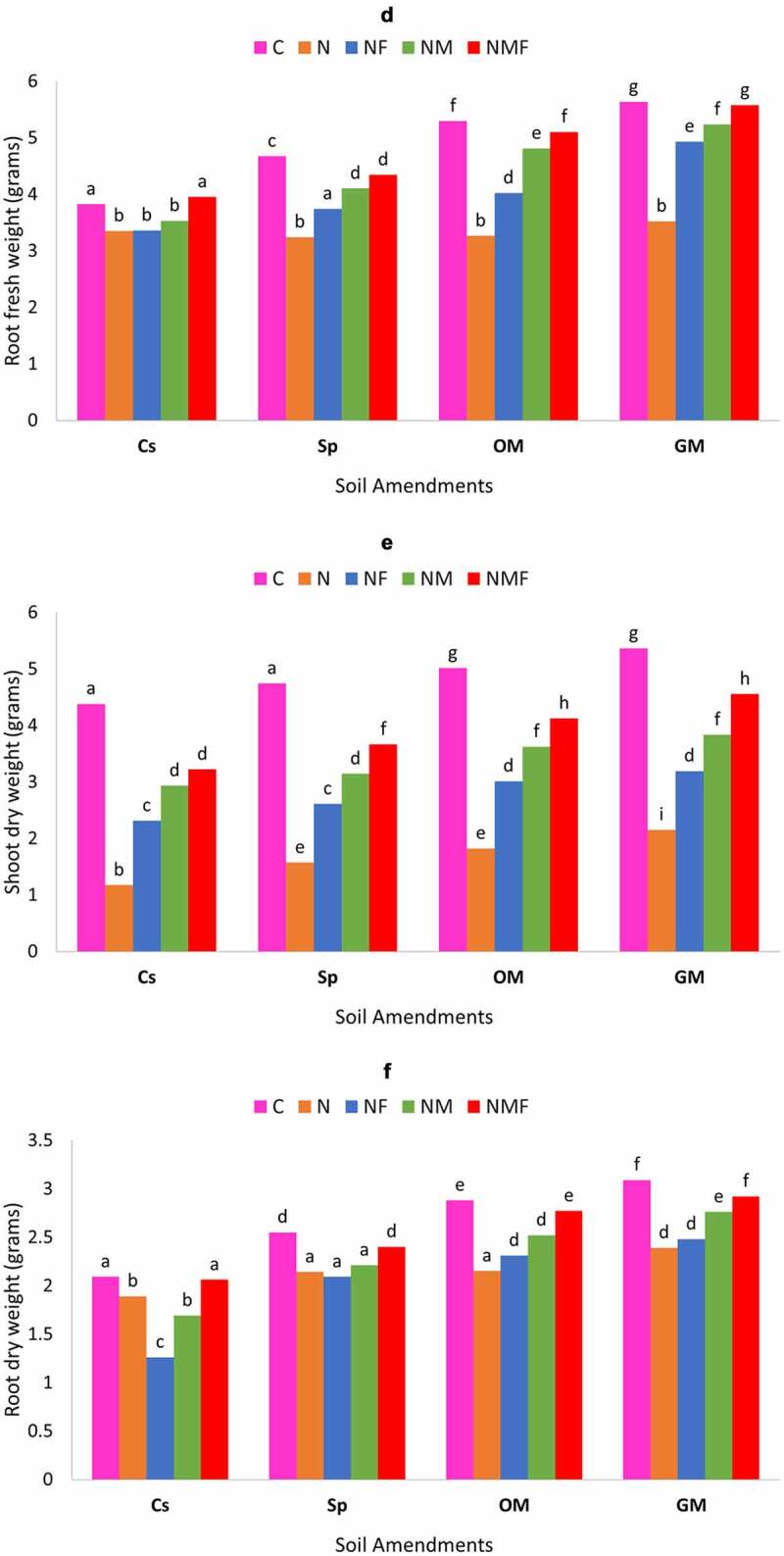
(Continued).

**Figure 4.**
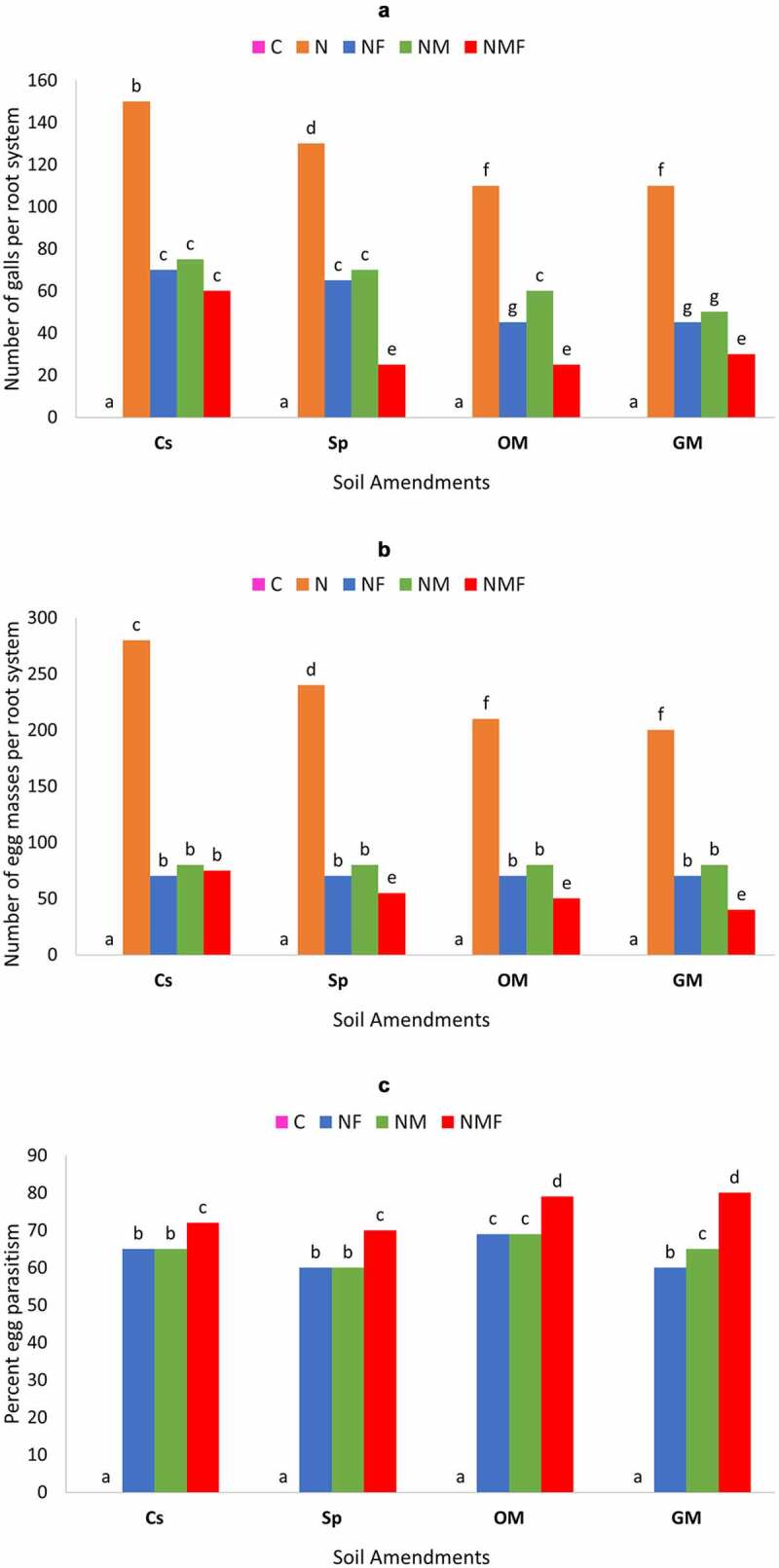
Effect of different treatments and soil amendments alone or in combination on number of galls per root system (a), number of egg masses per root system (b), percent egg parasitization (c) and fruit yield (d) of *C. annuum* plants infected with *M. incognita*. Within columns values of each parameter with the same superscript are not significantly different at (*P* = 0.05) Duncan’s multiple range test (DMRT). Soil amendments, Cs – Control Set, Sp – Superphosphate, GM – Green Manure, OM – Organic Manure, and soil treatments, C – Control, N – *M. incognita*, NF – *M. incognita* with *P. lilacinus*, NM – *M. incognita* with *P. lilacinus*, NMF – *M. incognita* with *R. fasciculatum* and *P. lilacinus.*

**Figure 4.**
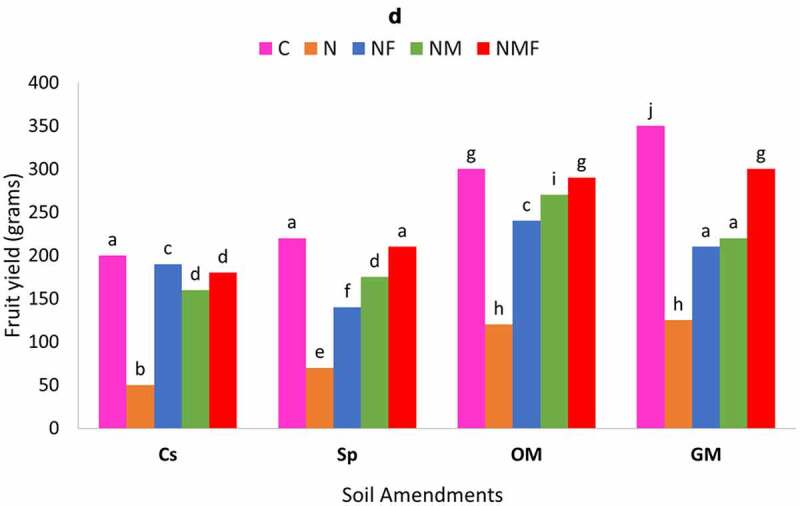
(Continued).

## Discussion

Due to their nematophagous properties, *Paecilomyces* spp. have been used in a variety of biological formulations in agricultural crop protection [27]. Among them, the egg parasite *P. lilacinus* potentially acts as a nematicidal agent, particularly against *M. incognita* as it drastically suppresses the growth and multiplication of this root gall forming nematode [28–32,]. The mycorrhizal fungi, which could colonize more than 90% of terrestrial plant species, have also been found to have a profound effect on reducing growth and proliferation of plant pathogens, especially root borne pathogens like *M. incognita* [16]. In the present study, the egg parasite, *P. lilacinus* and obligate root symbiont, *R. fasciculatum* alone or in combination effectively reduced the proliferation of root-knot nematode, *M. incognita* in the crop plant *C. annuum*. The individual inoculation of *C. annuum* with *P. lilacinus* (NF) remarkably reduced the number of galls and egg masses/root system in *M. incognita*-infected soil, thereby reducing the plant damage due to *M. incognita* (N). Results of present experimentation corroborate the findings of previous studies in black pepper and tomato revealing that the reduction in the root-knot population attributes to the fungal attack of larvae, or causing death of females before egg laying, thereby reducing their fecundity and rapid colonization and destruction of eggs formed [33,34]. The characterization of infection-related enzymes in tomato plants further revealed the effectiveness of *P. lilacinus* in controlling *M. incognita* [35–38].

An increase in the number of galls and egg masses/root system in the plants amended with superphosphate in comparison to the control set plants suggests a correlation between phosphate and root-knot development and vindicate the assumption that the higher P-level increases root-knot development and egg production thereby enhancing susceptibility of hosts to the nematode attack [39]. In the present study, not much difference was recorded among superphosphate, organic and green manure amendments; indeed, there was significantly lesser number of galls and egg masses/root system in the dual inoculated (NMF) or single inoculation of *C. annuum* plants with either of the two fungi (NF and NM). The two fungi drastically reduced the number of galls and egg masses of nematodes/root systems in comparison to that of the *M. incognita* (N) infected plants. Significantly lesser galls/root system in the roots of plants colonized by mycorrhizal fungi indeed concurs with the studies done on wheat and tomato [35,40]. The role of phosphate in mycorrhizal-nematode interaction remains incompletely understood thus requires additional research. The present study suggests that increased superphosphate in soil along with *P. lilacinus* and *R. fasciculatum* had a positive role against nematode *M. incognita* [41].

Inoculation of *C. annuum* with *R. fasciculatum* indeed reduced the effect and severity of *M. incognita* more than that detected in the case of *P. lilacinus* when used alone, attributing to the lower plant damage. Mycorrhizal symbiosis significantly improved root length and shoot length and their biomass in *M. incognita* infected soil. The mycorrhiza-mediated alteration in root morphology and increased plant growth under stress conditions is a well-established characteristic phenomenon of arbuscular mycorrhiza, which has been reported in several plants [16,42]. Increased root growth, particularly elongation zone and lateral root formation sites are prone to be penetrated by sedentary parasites that may be due to leakage of root exudate in these areas, however increased root growth in fungi-inoculated *M. incognita* infested plants did not show susceptibility to nematode infection in the present study. The development and reproduction of nematodes was indeed inhibited in the mycorrhizal roots in comparison to non-mycorrhizal roots, which may be due to a nonspecific defense response [43] and alteration of root morphology and physiology by mycorrhizal symbiosis [44]. Mycoparasitism potential of arbuscular mycorrhizal fungi remains unclear, but indirect effects of mycorrhizal symbiosis on the root parasites have been proposed as well. Mycorrhizal fungi penetrate plant roots through root hairs and make arbuscules and vesicles in the cortical cells and help the host plant in acquiring mineral nutrition from soil, in turn, obtain photosynthate/food from host plant. Similarly, M. *incognita* also depends for food and shelter on their host plant; therefore, the competition between these heterotrophs for carbon source, space and indeed infection sites could limit *M. incognita* population growth in the mycorrhizal plants, and seems a potential arbuscular mycorrhiza-mediated biocontrol mechanism [45].

In the present study, different soil amendments were applied and it was observed that the growth of *P. lilacinus* (in terms of CFU/g) was higher in plants amended with green and organic manure as compared to superphosphate and control sets that underlines the importance of soil amendment and *P. lilacinus* against root-knot nematode. The performance of *P. lilacinus* further increased when present in combination with *R. fasciculatum* (NMF), may be due to synergistic effect of both the fungi, thus controlling the infection and reducing the ability of *M. incognita* to reproduce in soil, which could further improve with soil amendments [46]. The individual inoculation either with *P. lilacinus* or *R. fasciculatum* significantly enhanced the growth of *Capsicum* plants and suppressed the nematode population in both plant roots and soil in all soil amendments, however, the combined inoculum of two fungi was more effective in the suppression of nematode infection [47].

The inoculation of plants with fungi along with organic matter amendments have shown significant reduction in gall development and reproduction of *M. incognita* [19]. Present study showed that *P. lilacinus* proliferates more extensively on nematode damaged roots, may be due to excessive leakage of root exudates from the galled roots, and the presence of egg mass on the root surface. This direct relation between leakage of nutrients from the roots and the ability of the fungus to colonize rhizosphere and rhizoplane has also been studied in tomato [48], which could be a great advantage for preventing the growth and development of plant parasites, thus signifying *P. lilacinus* as a biocontrol agent [49]. Such a relationship could decrease dependency on other soil factors and therefore increase the reliability of *P. lilacinus* as a biocontrol agent [8].

Adult female nematodes preparing for egg production require a considerable amount of nutrients, thus competing with the host for the pool of nutrients in the roots. The increased metabolic activity of massive cells of roots on which the nematode feeds stimulates mobilization of photosynthate from shoot to root tissues [50]. The mobilization and accumulation of substances reaches a maximum when the adult females commence egg laying and declines thereafter. In this study, despite comparable colonization of *P. lilacinus* in the rhizosphere (in terms of CFU/g) of NF- and NMF-treated plants, the percent egg parasitization by *P. lilacinus* was different. A differential percent egg parasitization between NF and NMF treatments can be substantiated by the above hypothesis. Greater egg parasitization in NMF-treated plants may be due to smaller size gall formation, which in turn could be attributed to the cumulative inhibitory effect of *R. fasciculatum* and *P. lilacinus* on the development and reproduction of *M. incognita*. There are various reports revealing the influence of mycorrhizal fungi on their host plants grow under nematode infested soils [36,39,51]; however, the presence of *R. fasciculatum* and *P. lilacinus* alone (NF and NM) did not result in a significant egg parasitization except in case of GM amendment.

In conclusion, the effect of two fungi in association with soil amendments revealed the potential applications of *R. fasciculatum* and *P. lilacinus* along with the supplementation of soil with green manure to control the growth and infection of *M. incognita*, and thereby the damage caused to vegetable crop plant like *C. annuum*.

## Data Availability

All data generated or analyzed during this study are included in this published article.
